# Solitary urothelial carcinoma penile metastasis on ^18^F–FDG PET/CT

**DOI:** 10.1016/j.radcr.2026.08.015

**Published:** 2026-09-05

**Authors:** Julian Svasti, Yann Leng Paul Chong, Andrew Russon, Victor Mansberg, Jeffrey Chen, Robert Mansberg

**Affiliations:** a Department of Nuclear Medicine and PET, Nepean Hospital, Kingswood, New South Wales, Australia; b I-MED Radiology Network, Miranda, New South Wales, Australia; c Faculty of Medicine and Health, University of Sydney, New South Wales, Australia

**Keywords:** Urothelial Cancer, Penile metastasis, ^18^F-DCFPyL PET/CT

## Abstract

Penile metastasis from other primary sites is rare. A 78-year-old man was referred for an ^18^F–FDG PET/CT scan for evaluation of perineal pain on a background of previous cystoprostatectomy 4 years previously for infiltrating urothelial carcinoma of the bladder. The scan demonstrated a hypermetabolic focus at the ventral aspect of the proximal penile shaft with urethral and percutaneous histopathological confirmation of penile metastasis from primary high-grade urothelial carcinoma. The patient subsequently underwent urethrectomy.

## Introduction

**Fig. 1:**
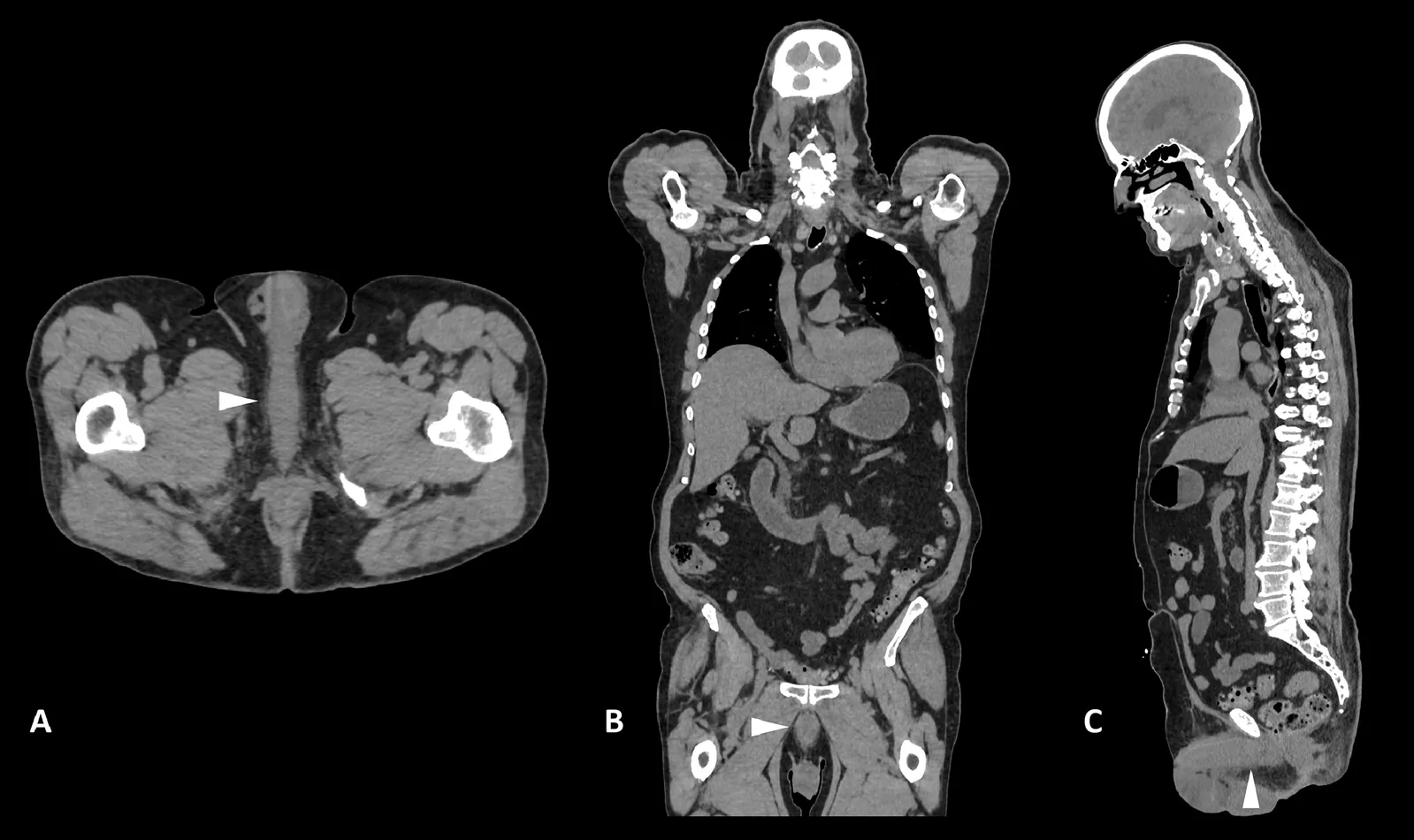
**A)** Axial **B)** Coronal **C)** Sagittal diagnostic CT images did not reveal a lesion to explain the symptoms.

A 78-year-old man was referred for an ^18^F–FDG PET/CT scan. He presented with severe perineal pain on the background of previous cystoprostatectomy for multifocal infiltrating urothelial carcinoma of the bladder 4 years prior. A cause for the symptoms was not evident on diagnostic CT (Fig. 1).

## Results

**Fig. 2:**
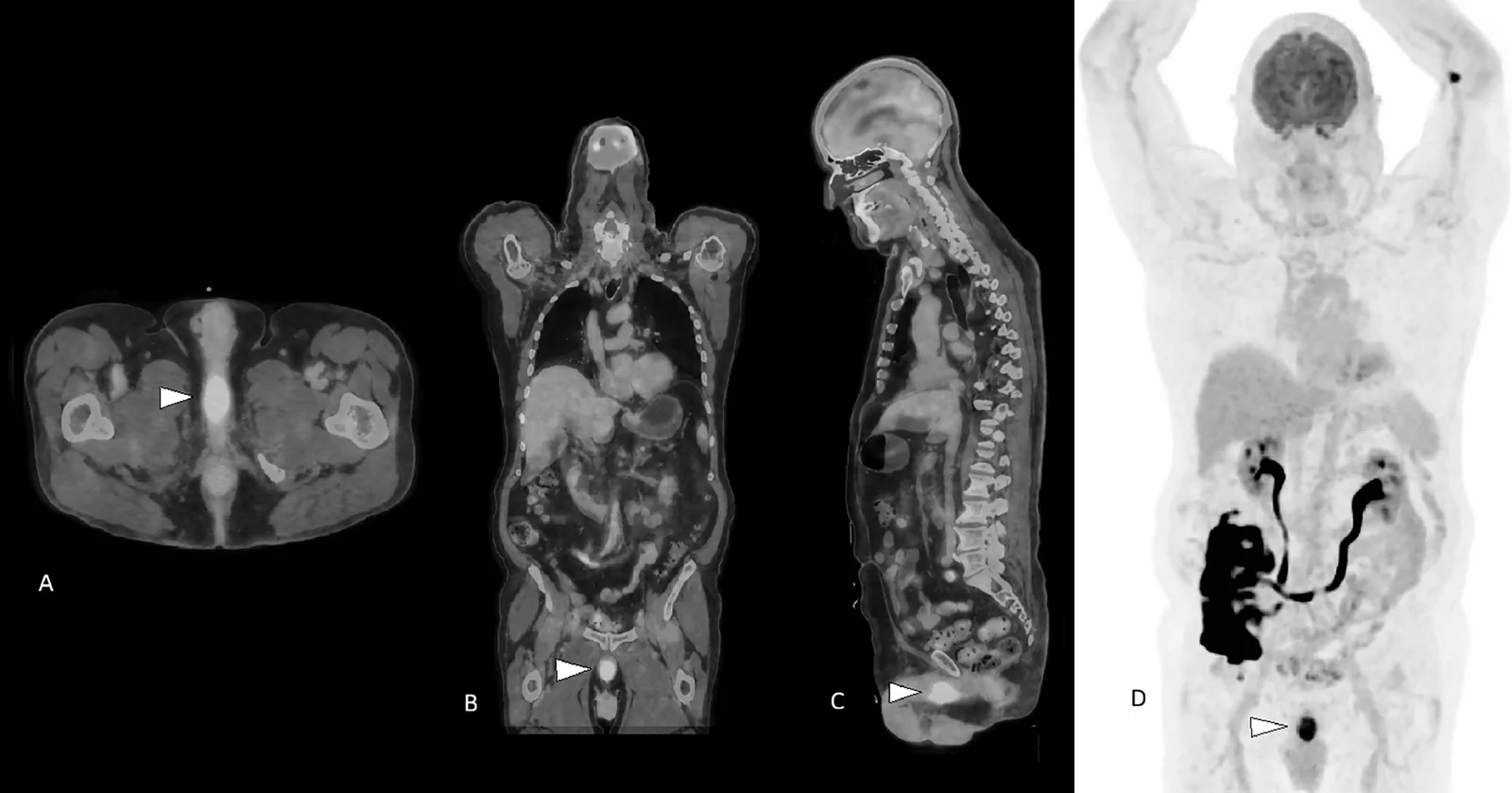
**A)** Axial, **B)** Coronal and **C)** Sagittal fused ^18^F–FDG PET/CT images, together with D) maximum intensity projection (MIP) PET image, demonstrated intense focal FDG uptake at the ventral aspect of the proximal penile shaft (arrowheads), corresponding to a subtle soft tissue abnormality on CT. No FDG-avid pelvic or retroperitoneal lymphadenopathy, or distant metastatic disease, was identified. Subsequent urethral and percutaneous biopsy confirmed invasive high-grade urothelial carcinoma, consistent with penile metastasis from the patient's previous bladder urothelial carcinoma. Definitive urethrectomy subsequently confirmed invasive pleomorphic high-grade urothelial carcinoma.

**Fig. 3:**
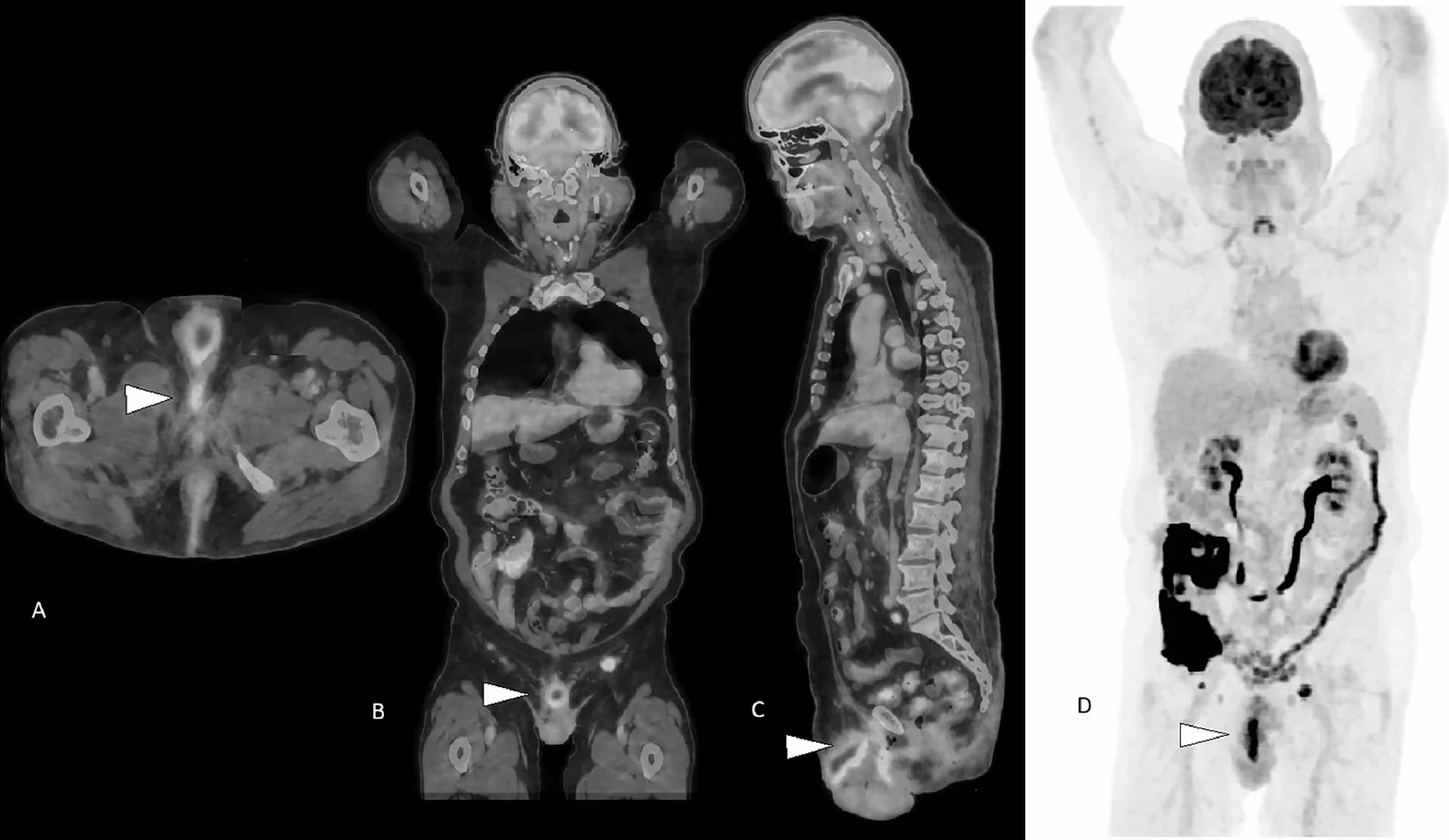
**A)** Axial, **B)** Coronal and **C)** Sagittal fused ^18^F–FDG PET/CT images, together with **D)** maximum intensity projection (MIP) PET image, obtained 6 weeks following urethrectomy, demonstrated persistent intense FDG uptake adjacent to the inferior margin of a postoperative fluid collection within the urethrectomy bed (arrowheads). The findings favoured post operative inflammatory activity, confirmed by repeat biopsy showing granulation tissue and chronic inflammation. ^18^F–FDG PET/CT also demonstrated the development of FDG-avid bilateral inguinal lymph nodes, more pronounced on the left, consistent with regional nodal metastatic progression. No distant FDG-avid metastatic disease was identified.

**Fig. 4:**
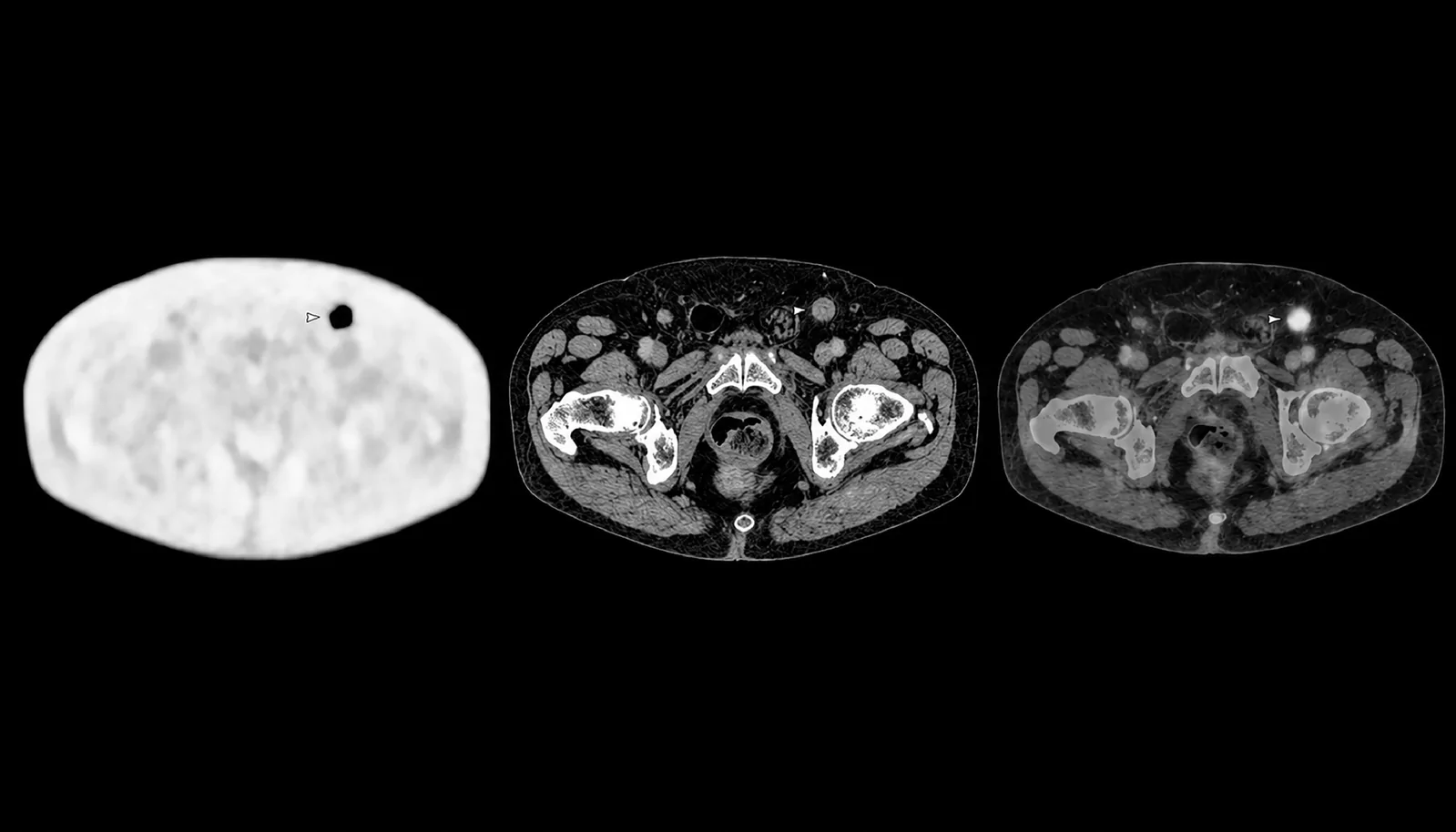
Axial PET, CT and Fused PET/CT images demonstrating FDG avid left inguinal lymphadenopathy (arrowheads).

Initial ^18^F–FDG PET/CT demonstrated an isolated intensely FDG-avid lesion involving the ventral aspect of the proximal penile shaft without evidence of regional nodal or distant metastatic disease (Fig. 2). Urethral and percutaneous biopsy confirmed invasive high-grade urothelial carcinoma consistent with penile metastasis from the patient's previous bladder urothelial carcinoma, and definitive urethrectomy subsequently confirmed invasive pleomorphic high-grade urothelial carcinoma. Subsequent^18^F–FDG PET/CT demonstrated progressive disease (Fig. 3), with interval development of FDG-avid bilateral inguinal nodal metastases, predominantly involving the left inguinal node (Fig. 4). Persistent intense FDG uptake adjacent to the postoperative urethrectomy bed demonstrated chronic inflammation on biopsy. No distant FDG-avid metastatic disease was identified.

## Discussion

Penile metastatic disease is rare despite the penis being highly vascularized. It most commonly arises from the genitourinary tract, particularly the bladder and prostate, followed by rectosigmoid origin and carries a poor prognosis [1]. Bladder cancer on the other hand is relatively common, being the 9th most common worldwide [2], of which urothelial cancer accounts for 90% of cases [3]. However, penile metastases from bladder urothelial carcinoma remains rare, with only several hundred cases reported [4]. Penile metastases can be asymptomatic or manifest with secondary priapism (20–50%) [5], perineal pain, ulceration, oedema, penile nodules, dysuria, hematuria and urinary retention [6]. Other more common sites of metastasis include skeletal, lungs, liver and peritoneum [7].

With regards to the distribution of metastases within the penis, the most common sites are the corpus cavernosa and glans, although tumors may also spread to the corpus spongiosum, urethra and penile skin [[8], [9]]. Several proposed mechanisms for metastasis exist. In our patient, with pre-operative biopsy proven multifocal urothelial carcinoma including at the prostate bladder neck, direct extension and intraluminal seeding/implantation are likely. Direct extension occurs when bladder cancer, particularly those involving the bladder neck or prostatic urethra, directly invades along the continuous urothelium which extends to the proximal urethra, and subsequently into the penis. Intraluminal seeding/implantation occurs when cancer cells are shed in the urine and implant along the urethral mucosa or are seeded during procedures. A key differential to consider is a metachronous primary arising from the penile urethra; the field cancerization theory posits that the entire urothelium is exposed to carcinogens which give rise to multifocal urothelial carcinomas. Other important mechanisms include retrograde venous flow, direct lymphovascular invasion and arterial spread [1,10].

^18^F-fluorodeoxyglucose (^18^F-FDG) is a positron-emitting radiotracer that acts as a glucose analog which utilizes altered glucose metabolism to localize tissues with a variety of physiological and disease processes. Its utility in staging has been questioned over high urinary excretion activity masking FDG accumulation of bladder carcinomas, with suboptimal sensitivity for locoregional disease evaluation. Nevertheless, it has emerged as an effective tool for evaluation of distant metastasis and treatment response, and the above-mentioned concerns can be partially mitigated by early diuresis or delaying acquisition time to facilitate identification of the primary bladder cancer and extravesical spread [9,11,12].

Penile metastases of any primary carry a poor prognosis, with an estimated median cancer-specific survival time of 14.5 months [13]. They are also often associated with disseminated disease and respond poorly to chemotherapy [9]. Treatment thus typically focuses on palliative strategies such as pain management, palliative radiotherapy/chemotherapy and surgical intervention such as penectomy [1].

Although penile metastasis from urothelial carcinoma is rarely reported, it is important to consider it as a differential in patients with a relevant history. ^18^F–FDG PET/CT remains an important tool for staging and evaluation of treatment response.

## Patient consent

The authors confirm that written, informed consent for publication of this case was obtained from the patient. This is retained with our records.
